# A combined experimental and density functional theory study of metformin oxy-cracking for pharmaceutical wastewater treatment†

**DOI:** 10.1039/c9ra01641d

**Published:** 2019-05-01

**Authors:** Ismail Badran, Abdallah D. Manasrah, Nashaat N. Nassar

**Affiliations:** Department of Chemical and Petroleum Engineering, University of Calgary 2500 University Drive NW Calgary Alberta Canada T2N 1N4 nassar@ucalgary.ca +1 403 210 3973 +1 403 210 9772; Department of Chemistry, An-Najah National University Nablus Palestine

## Abstract

Pharmaceutical compounds are emerging contaminants that have been detected in surface water across the world. Because conventional wastewater treatment plants are not designed to treat such pollutants, new technologies are needed to degrade and oxidize such contaminants. The newly developed oxy-cracking process was utilized to treat the antidiabetic drug, metformin. The process, which involved partial oxidation of metformin in alkaline aqueous medium, proved to decompose the drug into small organic molecules, with minimum emission of CO_2_, therefore, increasing its biodegradability and removal from industrial treatment plants. The reaction gaseous products were probed by online gas chromatography. The liquid phase before and after oxy-cracking was analyzed for total carbon content by TOC and gas chromatography mass spectrometry. The products formed from the nitrogen-rich drug included ammonia, amines, amidines, and urea derivatives. A reaction mechanism for the oxy-cracking process is proposed. Because the hydroxyl radical (˙OH) is believed to play a central role in the oxy-cracking process, the mechanism is initiated by ˙OH attacks on metformin, followed by single decomposition or isomerization steps into stable products. The reactions were investigated using density functional theory calculations and validated using high quality 2^nd^ order Møller–Plesset perturbation theory energy calculations.

## Abstract 2

Pharmaceutical compounds are emerging contaminants that have been detected in surface water across the world.

## Graphical abstract

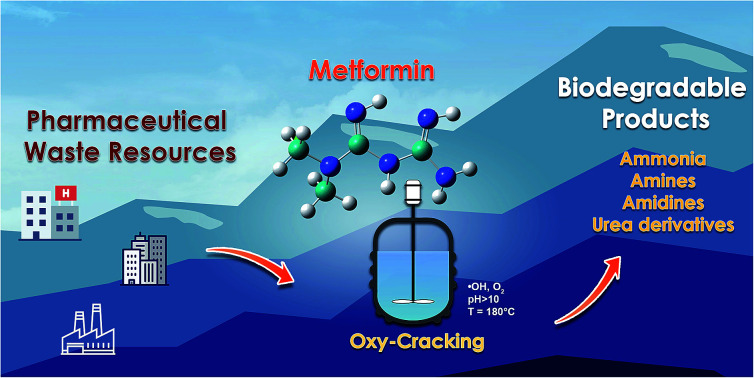

## 1 Introduction

The demand for pharmaceutical drugs and personal care products is on the rise, and there is a growing concern about their occurrence in surface water resources.^1–4^ Pharmaceuticals are one example of emerging pollutants that have found their way into fresh water resources through factories, hospitals, humans, livestock, and wastewater treatment plants (WWTPs). Because current WWTPs are not well designed to eliminate these kinds of pollutant, it is estimated that half of the pharmaceutical wastewater produced worldwide is released without any treatment, and, therefore, WWTPs are their main source.^1,3–5^ Pharmaceuticals are resistant to elimination because of their complex structures, high polarity, and low biodegradability, compared to natural organic pollutants.^1^ Because of their seepage into fresh water resources, pharmaceuticals have been detected in water bodies in low concentrations ranging from ng L^−1^ to several µg L^−1^.^4^ The low concentration and the nature of these pollutants have raised a debate on whether their water presence is harmful to humans and the environment. While some parties believe that such concentrations are below toxic levels, many studies argue that the combined effects of pharmaceutical mixtures are unknown and must not be ignored.^2,3,6–8^

Numerous studies showed that the presence of pharmaceutical compounds in the environment has negative effects on both terrestrial and aquatic life. These effects include bacterial resistance to antibiotics, potential toxicity of chemical combinations, allergic complications, behavior and migratory patterns fertility in fish.^2,7,9^ In addition, there is increasing concerns on the endocrine disruption caused by these pollutants.^2,6,8,10,11^ That is the perturbation of endogenous hormone function by external chemicals, such as pharmaceuticals. Based on this, there is a need to seek new technologies to treat pharmaceutical compounds and personal care products in WWTPs.

The most common technologies to treat wastewater in WWTPs are coagulation/flocculation, filtration, adsorption, and advanced oxidation processes (AOPs).^4,5^ These methods are not fully efficient in removing pharmaceutical compounds. Besides, applying these techniques involve high operation costs and lead to the formation of by-products and concentrated residues. Coagulation and adsorption techniques do not completely eliminate pharmaceuticals presence in wastewater resources. AOPs, in the other hand, operate for high and low concentrated samples and, typically, leads to fully photodegrade the contaminants and produce significant amounts of CO_2_, or other harmful gases. Therefore, a combination of techniques or developing new ones is required and of high amount importance.^2,4,5^

Recently, our research group has employed a new technique that combines oxidation and cracking reactions (oxy-cracking) at mild temperatures and pressures in an alkaline medium, whereby heavy organic compounds are converted into simple molecules with minimum emissions of CO_2_.^12,13^ The technique was successfully applied on residual feedstocks like petroleum coke and asphaltenes for converting them to value-added products like humic acid analogs.^12,13^ We have recently applied this technique for enhancing settling and dewatering in oil sands tailings.^14^ In the oxy-cracking process, residual bitumen and hydrocarbons are cracked and solubilized in the liquid phase, freeing the fine particles to agglomerate and settle faster.^14^ Hence, the aim of this study is to apply the oxy-cracking technique on pharmaceutical organic contaminants to convert them into small fragments and intermediates, thus improve their biodegradability, with minimum emissions of harmful gases, like CO_2_.

In their review on the health hazards of pharmaceutical compounds, Fent *et. al.*, classified those compounds with their high environmental impacts to have (1) high production volume; (2) environmental persistence; and (3) high biological activity.^8^ The anti-diabetic drug, metformin, appears to be a potential candidate to meet this classification. Metformin (*N*,*N*-dimethylbiguanide), abbreviated hereinafter by MF, is widely prescribed for type 2 diabetes. Despite its popularity, MF cannot be metabolized by the human body and it is estimated that 70% of this drug is excreted in the urine and faeces.^15–17^ For this reason, considerable amounts (up to 100 ppm) of the drug was detected in WTTPs effluents in many places worldwide.^15,18,19^ Recent studies showed that MF was proven to pose ecological risks to aquatic life with concentration levels close to those detected in surface water. It was also reported that farther transformation of MF into guanylurea might pose “unknown effects” on human health.^16^ Impact of MF on fish was proven recently in many studies.^10,11,20^ It was shown that MF at concentrations in the range of what was detected in surface water and WWTPs effluents cause aggressive behavior and endocrine disruption in fish.^10,20^

This work presents the first application of the oxy-cracking technique to thermally decompose MF in an aqueous medium aiming to enhance its biodegradability. The work also targets exploring the potential of converting nitrogen-containing waste into commodity chemicals. Since MF is a nitrogen-rich compound, it is expected to form light nitrogen products such as ammonia and amines.^17^ Herein, the oxy-cracking reaction took place in a Parr reactor containing alkaline solution (pH > 8). The reaction products were probed by GC, TOC, and GCMS. To further understand the reaction mechanism of MF oxy-cracking, theoretical calculations were performed to explore the reaction pathways of MF with ˙OH radicals, which is believed to play the central role in oxy-cracking.^13^ Geometry optimization of all species in this work was accomplished by density functional theory (DFT) methods. The 2^nd^ order Møller–Plesset perturbation theory (MP2) was also employed to compute the final energies of optimized species.

## 2 Experimental

### 2.1 Materials

*N*,*N*-Dimethylbiguanide hydrochloride (95.0%), commonly known as metformin, was purchased from a local pharmacy in Calgary, Alberta, Canada. Potassium hydroxide (KOH, ACS reagent, ≥85%, Sigma-Aldrich, Ontario, Canada) was used to provide the alkaline medium. Organic solvents such as dichloromethane, methanol, and ethanol, all of ACS grade, were purchased from Sigma-Aldrich, Ontario, Canada and used as received. O_2_, used for the oxy-cracking experiments, N_2_ and air mixture, used for the TOC analysis, and helium, the carrier gas for the GCMS, were all of ultrahigh purity grade, obtained from Praxair, Calgary, Canada.

### 2.2 Oxy-cracking experiments

The experimental setup used in this study was described in details in previous publications.^12,13^ Briefly, the experiments were performed in a 100 mL stainless steel reactor vessel (model number 4598, Parr Instrumental Company, Moline, Il, USA), equipped with a heating oven connected to temperature controller. Type K thermocouple was used to read the temperature. The pressure inside the reactor was controlled and monitored using a pressure gauge. The reactor vessel is capable of handling pressures up to 1700 psi and temperatures up to 270 °C. In a typical experiment, 30 mL of 16 g L^−1^ MF solution were added to the reactor along with 0.2 g KOH. The solution was kept alkali to assist in solubilizing the reaction products and minimize corrosion. The reactor was filled with 750 psi of O_2_ gas, after few flushes with pure O_2_ to remove any traces of air. The reactor vessel was leak tested by sealing and pressurizing the vessel with O_2_. The reaction was then started by heating the mixture to the desired temperature and taking the zero time to be that of reaching that temperature. The experiments were performed at 15, 30, 60, and 120 min. Once the reaction is complete, the reactor was cooled down to room temperature and the gaseous products were analyzed using gas chromatography (GC, SRI 8610C, SRI Instruments). The GC was equipped with two packed columns connected in parallel; a 3′ molecular sieve and 6′ Hayesep-D columns. The molecular sieve column was used for permanent gases, while the second was used to detect hydrocarbons up to five carbon atoms. Helium gas was used as a carrier. The liquid phase was analyzed by total carbon content (TOC) and gas chromatography mass spectrometry (GCMS). Also, a sample of the headspace was captured in a lecture bottle for further GCMS analysis.

### 2.3 Total organic carbon (TOC) analysis

The carbon content of the organic and inorganic materials present in the reaction mixture was analyzed using total organic carbon analyzer (Shimadzu, TOC-L CPH/CPN). After separating the liquid phase as described before, diluted samples in deionized water were analyzed for their total carbon (TC), total organic carbon (TOC), and inorganic carbon (IC). Both TC and IC measurements were calibrated using standard solutions of potassium hydrogen phthalate and sodium hydrogen carbonate. More details on the TOC analysis and calibration can be found elsewhere.^12,13^

### 2.4 GCMS analysis

The oxy-cracked reaction mixture was extracted multiple times using dichloromethane in a 100 mL separatory funnel. Both the aqueous and the organic phases were then analyzed by gas chromatography-mass spectrometry instrument (GCMS, QP5000, Shimadzu) equipped with AOC-20i autosampler. A general purpose 5% polar column (Alltech Heliflex® AT™-5ms, 30 meter) was used to separate the products. In a typical analysis, 2.0 microliter of 1% diluted sample in dichloromethane was injected at 320 °C into the autosampler. Oven temperature was held constant at 35 °C for 3 min and ramped at 10 °C min^−1^ to a final temperature of 280 °C. The temperature was held again at 280 °C for 6 min. Helium (28.5 kPa) was used as a carrier gas at a flow rate of 1.5 mL min^−1^ with a split ratio of 1 : 10. The analysis was performed in electron ionization (EI) mode in the scan range 30–300 *m*/*z*, scan interval = 0.56 s, and scan speed = 500. NIST mass spectral library 2014 was used to help identifying the unknown components. Selected ion monitoring (SIM) mode was used to analyze the gaseous headspace of the reaction. Prior to each SIM analysis, the instrument was set to detect only the *m*/*z* peaks for ammonia, N_2_, CO_2_, in addition to mono- and dimethylamines. The lecture bottle containing the gas sample was connected directly into the injector through a split valve. A short burst of the sample was then injected by opening the valve for few seconds at 120 °C and at interface temperature of 280 °C. The oven temperature was then ramped from 30 °C to 180 °C at 10 °C min^−1^.

### 2.5 Theoretical calculations

Density functional theory (DFT) calculations were employed to construct the reaction mechanism for the oxy-cracking of metformin. Geometry optimization and frequency calculations were performed using the B3LYP functional^21^ along with the 6-31+G(d,p) basis set, which proved to be a good method to optimize similar systems.^13,17,22–24^ Transition states were discovered by exploring the potential energy surface (PES) along the desired reaction coordinate. Each transition state was confirmed to have one and only one imaginary frequency along the reaction coordinate, and an intrinsic reaction coordinates (IRC) was constructed to validate its authenticity. Zero-point energies (ZPE) were scaled by a factor of 0.9806 as suggested by Scott and Radom.^25^ The *singlet* ground state was assumed for all closed shell species unless mentioned otherwise. For open shell free radicals and intermediates, the unrestricted version of the functional, *i.e.*, uB3LYP, was used. Final energies were calculated using the 2^nd^ order Møller–Plesset perturbation theory (MP2)^26,27^ and the 6-311+G(d,p) basis set. The calculations of the thermochemical parameters; enthalpy, entropy, and Gibbs free energy involved in this study can be found in previous works.^22,23,28^ All reactants, intermediates, transition states and products were optimized to their equilibrium geometries using Gaussian 16 (ref. 29) and viewed using Gaussview.^30^

## 3 Results and discussion

### 3.1 Oxy-cracking of metformin

**Fig. 1:**
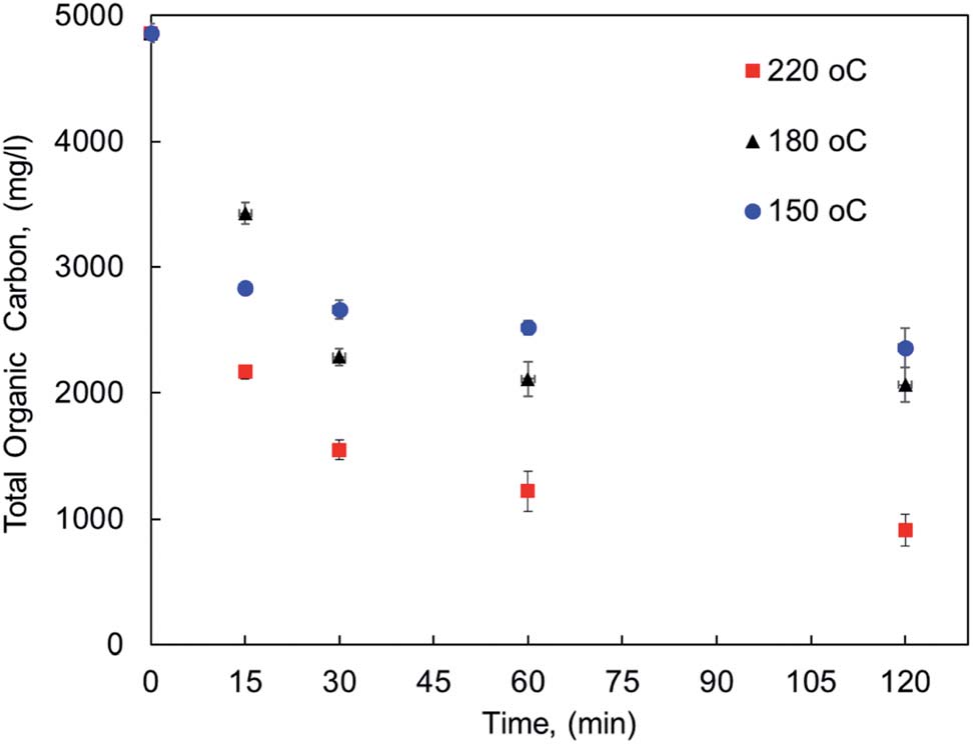
The concentration of pharmaceutical organic compounds in the liquid phase as lumped TOC at different reaction temperatures and times.

The effect of reaction conditions on MF oxy-cracking was investigated at different reaction temperature (150–220 °C) and times (15–120 min). To keep the oxy-cracking reaction in the liquid phase, a sufficient pressure was kept in the reactor, thereby preventing the liquid from vaporizing. This implies that the applied pressure must exceed the vapor pressure of the liquid solution at a given temperature. Therefore, in preliminary experiments, it was observed that there is no pressure effect on the oxy-cracking reaction when the oxygen pressure was beyond 750 psi, thus it was fixed to be 750 psi for all runs.^12,13^ Additionally, the mixing rate was set to 1000 rpm, thus enhancing the oxygen transfer from the gas phase to the liquid phase by minimizing the gas/liquid mass transfer resistance and also maintaining a relatively uniform concentration of MF content in the reactor.^12,13^ The effect of reaction temperature along with the reaction time on MF concentration as lumped TOC concentration (total organic carbon content) into the aqueous solution is presented in Fig. 1. The error bars in the figure represent the calculated standard deviation based on three TOC measurements. It is well known that the reaction temperature plays an important role in the wet air oxidation reaction. As reported elsewhere, the removal efficiency of TOC, pollutant disappearance, chemical oxygen demand (COD) abatement and biodegradability enhancement are very sensitive to the reaction temperature for wastewater treatment.^31–33^ However, the target here is different. As shown in Fig. 1, the lumped TOC values of both oxygenated hydrocarbons (the oxy-cracked MF) and the original pharmaceutical organic contaminants in the liquid phase are practically decreased with temperature with any considered residence time. Increasing the temperature (*i.e.*, up to 220 °C) the organic carbon content is continuing to decrease with time. For all temperatures, the decreasing in TOC tends to level off at 30 min of oxy-cracking reaction time. These findings support the concept of oxy-cracking reaction where all the oxygenated hydrocarbons are present in the liquid phase without a significant reduction in the TOC values during the reaction, thus avoiding CO_2_ formation. This can be rationalized by the fact that a large number of small-stable fragments and intermediates compounds were formed during the oxy-cracking reaction and such species are not impacted by the considered temperature.^34^ Another reason is due to the highly basic medium that might shorten the lifetime of the formed free radicals which is known to have scavenging effects.^12^

**Fig. 2:**
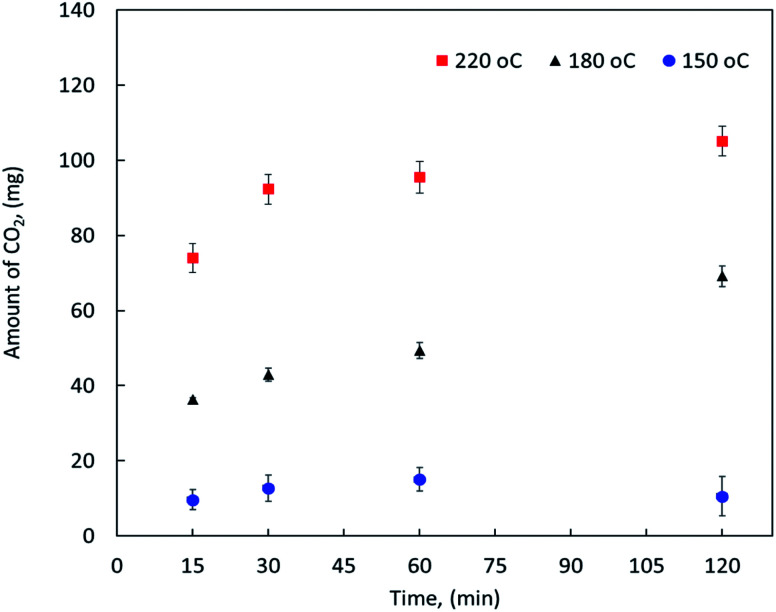
The amount of CO_2_ released in the gas phase as detected by GC at different reaction temperatures and times.

On the other hand, the effect of reaction temperature and time on the CO_2_ emission were also investigated during the MF oxy-cracking. It is worth mentioning that during the oxy-cracking experiments, neither carbon monoxide nor CH_4_ was detected in the gas phase using the thermal conductivity detector (TCD) connected with molecular sieve and Hayesep-D columns of our GC. Since the provided GC with TC detector is measuring the permanent gases and hydrocarbons up to C5, carbon dioxide was found to be the most detectable and dominant gas in this level of measurement. However, some of the produced gases might be transferred to the gas phase which were qualitatively detected using GCMS. Fig. 2 shows the amount of CO_2_ released in the gas phase at different temperatures of 150, 180 and 220 °C and different times of 15, 30, 60, and 120 min as detected by the online GC analyzer. As shown, the possibility of producing CO_2_ in the gas phase is increased with increasing the reaction temperature. At high reaction temperature (*i.e.*, 220 °C), the emission of CO_2_ is slightly increased with the reaction time. Even at the shortest reaction time of 15 min, the amount of produced CO_2_ is detectable by GC. Similar observations can be noted at a lower reaction temperature. However, low reaction conversion might occur at low temperature and short reaction time (*i.e.*, 150 °C and 15 min), this is because low emission of CO_2_ gas can be detected by GC at these reaction conditions. It can be observed here that the high reaction temperature is not favorable in the oxy-cracking process as combustion might be taken place. Therefore, the optimum operating conditions for the high degree of MF oxy-cracking has to be synchronized with minimal CO_2_ emission. Nevertheless, small amounts of produced CO_2_ may also be trapped in the aqueous basic solution (pH > 8) in the form of carbonates and bicarbonates.

Herein, the concept of the oxy-cracking reaction is to partially oxidize and crack the hydrocarbons into valuable products that have lower molecular weight than their starting materials, by incorporating oxygen and avoiding CO_2_ emission. Unlike the wet air oxidation process, which converts all hydrocarbons into CO_2_. Therefore, at certain oxy-cracking conditions, the starting material, MF in our case, do not completely oxidize to CO_2_ and other gases but instead form intermediate compounds and oxygenated hydrocarbons with different functionalities. As will be discussed in upcoming sections, these compounds were mostly amines, amidines, pyridines, and urea derivatives.

It is believed that the hydroxyl radical (˙OH) plays an essential role in the wet oxidation of hydrocarbons.^35–37^ In our previous work on the oxy-cracking of Quinolin-65 (Q65), an aromatic structure that mimics asphaltenes, we also showed that the reactions of ˙OH radical with Q65 can account for the overall reaction mechanism. Although oxidation by O_2_ or O atoms might also occur, it is agreed on that those with ˙OH radicals and hydroxide anions (OH^−^) play the most part, especially under basic conditions.^37–40^ Once the ˙OH radicals are generated, attacks on MF initiates a series of free-radical reactions that form different types of intermediates as well as stable products. These products were probed by GCMS analysis.

### 3.2 GCMS analysis of oxy-cracking products

**Fig. 3:**
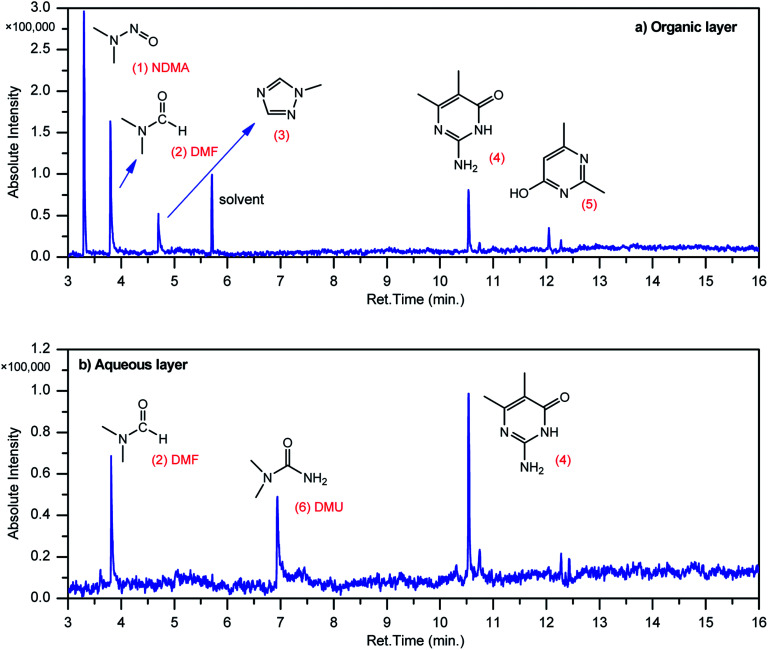
Total ion chromatograms (TIC) of (a) the organic layer oxy-cracked MF sample after extraction by dichloromethane, and (b) the aqueous layer after the same extraction, as obtained by GC-MS. Oxy-cracking took place at 150 °C, 750 psi of O_2_, for 2 h.

GCMS analysis was performed for all samples taken from the oxy-cracking experiments. Small varieties of organic products were detected from experiments performed at temperatures as low as 150 °C. An example of a GCMS chromatogram obtained from the organic layer after extraction with dichloromethane of the oxy-cracked MF is shown in Fig. 3a. Also, panel b of the figure shows the peaks obtained from the aqueous layer. The compounds corresponding to the numbered peaks are listed in Table 1. It is clear that more organic compounds were extracted to the organic layer than the aqueous phase. It is interesting to observe that only few organic materials were detected by GCMS. This reflects the efficiency of the newly developed oxy-cracking process in producing limited numbers of compounds, which could facilitate their separation in industrial plants. Our GCMS has detected mono and dimethyl amines as primary products. But since they are gases at room temperatures, they eluted early, and their peaks coincided with that of the solvent. Some other products were *N*-nitrosodimethylamine (NDMA), *N*,*N*-dimethyl formamide (DMF), *N*,*N*-dimethylurea (DMU), dimethylguanidine (DMG), and hydroxyacetonitrile. Some of these products are useful, such as DMF which is a common organic solvent, and DMU, DMG and dimethylcyanamide which are building blocks for many industrial and pharmaceutical applications. It is noteworthy to mention that DMG was reported to be a major product of the transformation of the MF during chlorine disinfection of water.^15,18^ Some of these products, unfortunately, are hazardous, such as hydroxyacetonitrile (known as glycolonitrile) and NDMA. The latter is a common by-product of many wastewater treatment processes.^41,42^ However, these products were found to have less promotion when they compared with the valuable products.

Compounds detected by GCMS as listed in Fig. 3

**Table:** 

No.	Compounds listed in Fig. 3
1	*N*-Nitrosodimethylamine (NDMA)
2	*N*,*N*-Dimethylformamide (DMF)
3	1-Methyl-1,2,4-triazole
4	1-Methyl-4-methylaminocytosine
5	2,4-Dimethyl-6-hydroxypyrimidine
6	*N*,*N*-Dimethyl urea (DMU)

**Table:** 

Other compounds detected by GCMS not shown in Fig. 3	
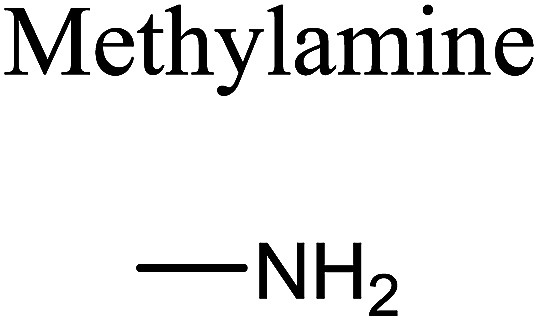	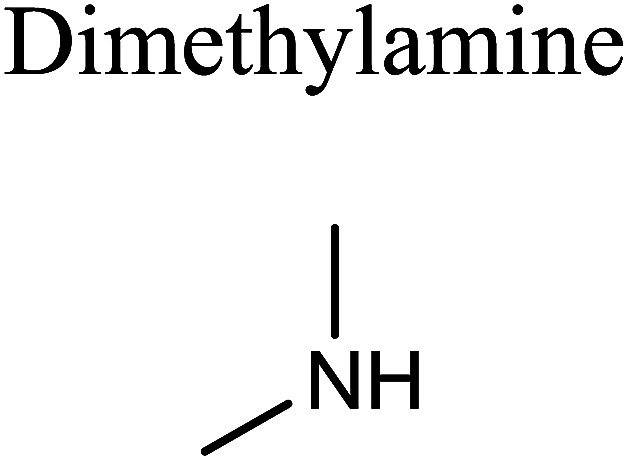
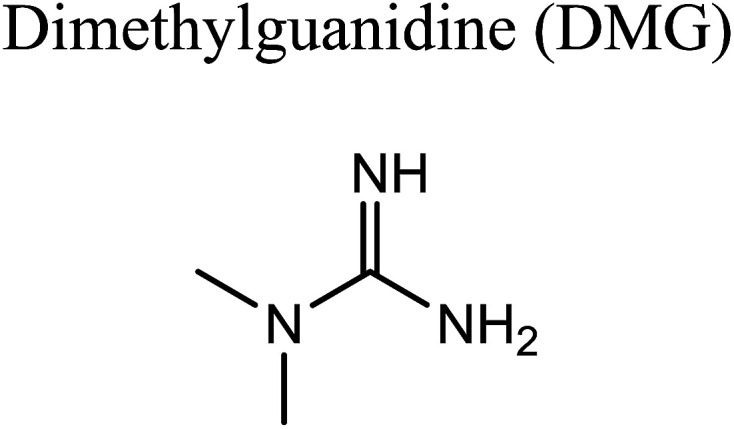	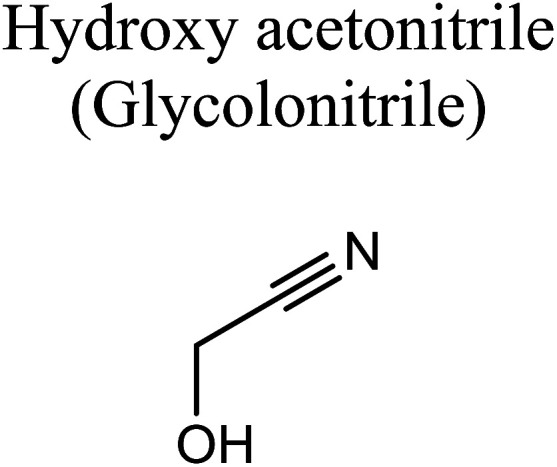
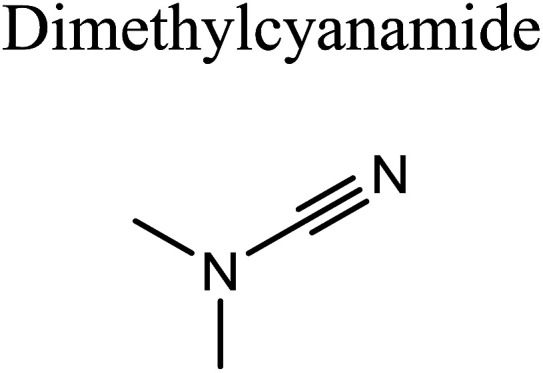	

As mentioned earlier, GCMS analysis was performed for all experimental samples. A qualitative evaluation of the produced products indicated that the number of products and their signal intensities were decreasing with the increase in the reaction conditions. At higher temperature (220 °C) and longer reaction time (2 h), combustion rate increases and drives more products to oxidize into CO_2_. This result is in concert with that obtained from the TOC analysis, where the dissolved carbon levels in the liquid phase slightly decreases as the reaction conditions become harsher.

Based on the previous discussion, it is evident that the oxy-cracking reaction is not very efficient at low temperature near 150 °C. On the other hand, at a high temperature of 220 °C and long reaction times, the reaction droves off more CO_2_ and less biodegradable intermediates, as shown in Fig. 2 and as confirmed by the GCMS analysis. Therefore, we concluded that the best operating conditions for the oxy-cracking of MF is at 180 °C and reaction time of 2 h.

In addition to the products mentioned in Table 1, ammonia was also detected by the GCMS. When a lecture bottle of the headspace of the oxy-cracked MF was analyzed using the selective ion monitoring (SIM) technique, mass peaks of ammonia, in addition to those of mono- and domethylamines, O_2_, H_2_O, CO_2_, and N_2_ were observed. Because of the excessive amount of oxygen found in the gaseous sample, it was difficult to quantify the amount of ammonia in the sample. Formation of ammonia from MF come as no surprise, since amidines, the class of chemicals that MF belongs to, form ammonia upon heating.^43–45^

As we already mentioned, one of the main goals of the oxy-cracking of MF is to enhance the biodegradability of MF and enhance its removal in WWTPs. The limited number of products and their simple structure helps accomplish this goal. Furthermore, observation of ammonia in this work drives us to think about oxy-cracking as a new technology to produce ammonia, which is considered to be the ‘renewable energy source’ of the future,^46^ out of nitrogen-containing waste.

### 3.3 Proposed reaction mechanism for the oxy-cracking reaction

**Scheme 1:**
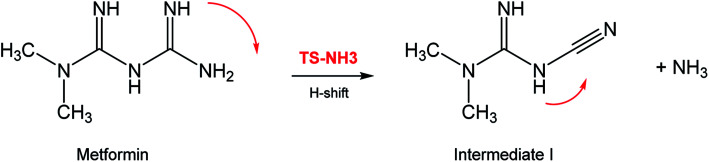
Formation of ammonia from metformin, by thermal 1,3-H-shift.

**Fig. 4:**
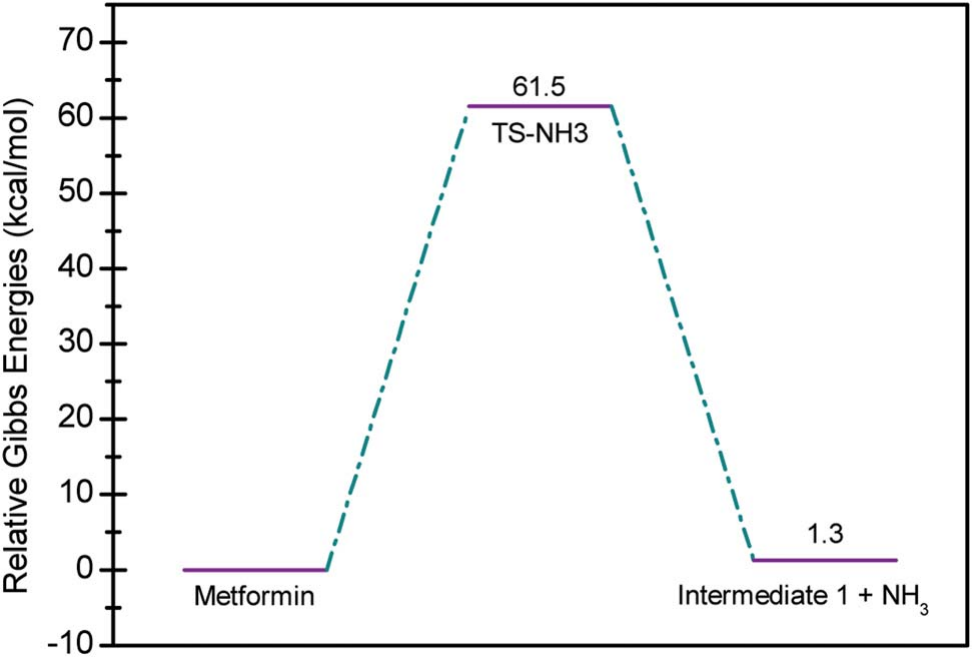
Potential energy level diagram for the 1,3-H shift reaction leading to the formation of ammonia from metformin. Energies represent relative Gibbs free energies in kcal mol^−1^ at 298 K and 1 atm (ZPE corrections are included).

In seeking a plausible reaction mechanism for the oxy-cracking of MF, we employed quantum theoretical calculations as described in the theoretical section. Such calculations allow us to explore different reaction pathways and, most importantly, locate the transition states connecting reactants to products, where such TSs are difficult to detect experimentally. The formation of ammonia from MF during oxy-cracking reaction can be first visualized simply as a 1,3-hydrogen shift from NH to the NH_2_ terminal in MF, as depicted in Scheme 1.^17^ Using the DFT functional, B3LYP and the 6-31+G(d,p) basis set, we located the TS for this reaction, and an optimized geometry for the TS and intermediate I are shown in Fig. S1 in the ESI.† We found the reaction barrier from MF to the TS to be 61.6 kcal mol^−1^. This value is in very good agreement with that obtained by Andrés *et. al.*, at HF/6-31G** for the 1,3-H shift of formamidine (62 kcal mol^−1^).^43^Fig. 4 shows a potential energy diagram for this reaction pathways. Although the reaction energy is slightly endothermic (1.3 kcal mol^−1^), the high barrier of *ca.* 62 kcal mol^−1^ allows this reaction to take place only at extreme conditions; or with the presence of a catalyst. In the subsequent section, we will show how reactions with ˙OH radical provide alternative and more favorable pathways to produce ammonia, and the other valuable products of the oxy-cracking reactions.

As mentioned in our previous work,^13,47^ the oxy-cracking reaction is initiated by free-radical species, such as ˙O, ˙HO_2_, or ˙OH, primary attacks on the organic molecule. This leads to a free-radical chain reaction to form further radicals. Depending on the reaction conditions, the reactions terminate at stable small products (or intermediates) or proceed for further oxidation. Thermal degradation, such as the case of Scheme 1, can also contribute to the formation of products. Our group recently reported an experimental and theoretical investigation of the thermal decomposition of MF in the absence of oxygen.^17^ Nevertheless, it is strongly believed that ˙OH radicals play the primary role in the oxy-cracking reaction, as reported elsewhere.^13,37,40,48,49^

**Scheme 2:**
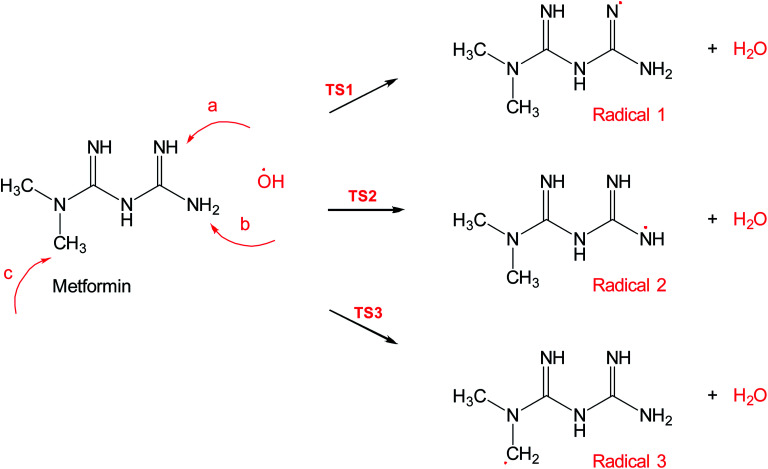
Reaction mechanism A, hydrogen abstraction by ˙OH radical in metformin.

**Fig. 5:**
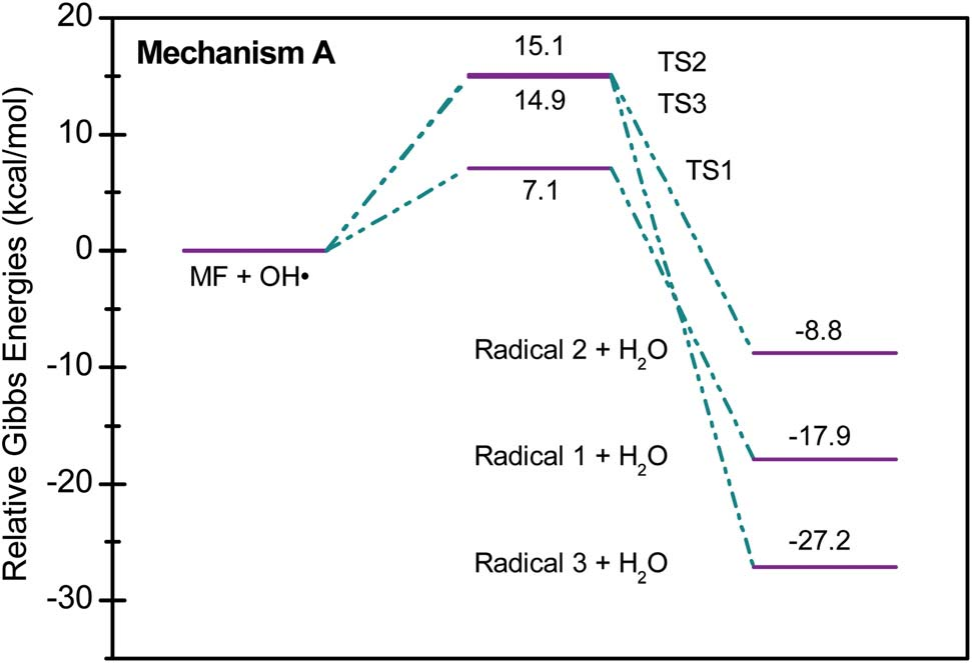
Potential energy level diagram for reaction mechanism A, hydrogen abstraction by ˙OH radical in metformin. Energies represent relative Gibbs free energies in kcal mol^−1^ at 298 K and 1 atm (ZPE corrections are included).

Scheme 2 shows three of the possible H-abstractions by ˙OH from the NH, NH_2_ terminals, and CH_3_ to form the free radicals, 1, 2, and 3, respectively. The product of this abstraction is water. The reason we studied these reactions is to understand the reactivity of ˙OH radical with MF. The transition states, TS1, TS2, and TS3, for the three reaction pathways, were all located successfully. The optimized geometries for the transition states and the free radicals involved in this mechanism are all shown in Fig. S2 in the ESI.† The activation barriers, in terms of free Gibbs energy (Δ*G*^‡^_298_) for these reactions were calculated to be 7.1, 15.1, and 14.9 kcal mol^−1^ at MP2/6-311+G(d,p//B3LYP/6-31+G(d,p) level of theory. Also, all H-abstractions were exergonic (Δ*G*_298_<0), as shown in Fig. 5. Surprisingly, these values are much less than those obtained for the H-abstractions from Q65.^13^ The Δ*G*^‡^_298_value for the H-abstraction in that case was estimated to be 134.4 kcal mol^−1^ at the same level of theory, and the reaction was endergonic (Δ*G*^‡^_298_ > 0) by 114.5 kcal mol^−1^. The difference in the energies is due to the nature of abstraction. In MF, the abstracted H was either an NH, NH_2_, or CH_3_ hydrogen in a small molecule, where in Q65, the abstracted H was an aromatic one attached to a large stable aromatic system. This infer that attacks of ˙OH radicals on nitrogen-rich amidines, such as MF, are highly favorable, even with no catalyst.

These theoretical findings support our perspective of oxy-cracking as an efficient technology to oxidize waste organic compounds. The condition of oxy-cracking; the moderate pressure of O_2_, moderate reaction temperatures, and the presence of KOH, allows the generation of ˙OH radicals, which are proven, using quantum theoretical calculations, to be very reactive against N-containing small molecules.

**Scheme 3:**
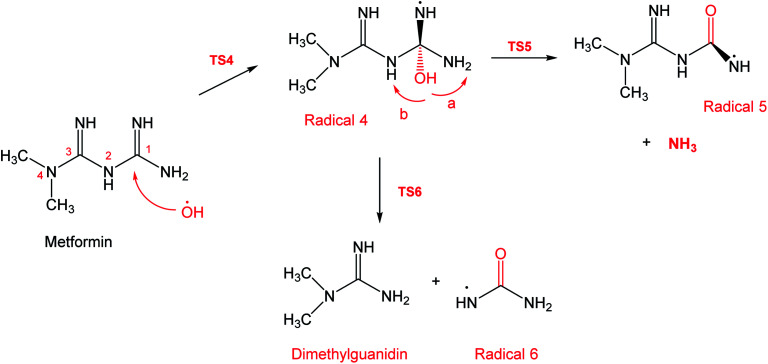
Reaction mechanism B initiated by ˙OH radical attack on C1 in metformin.

To account for the formation of some of the reaction products detected by GCMS, an initial attack of ˙OH radical on C1 of metformin was investigated. As illustrated in Scheme 3, the reaction starts with the formation of the free radical 4, which decomposes either to radical 5 and ammonia through TS5, or into dimethylguanidine (DMG) and radical 6, through TS6. Optimized geometries for all species of this mechanism are shown in Fig. S3.† Both NH_3_ and DMG were observed during the oxy-cracking experiments. However, this mechanism can account better for the formation of ammonia since the energies are much less than those obtained for the 1,3-H shift mechanism discussed earlier. As shown in Scheme 3, the first energy barrier (Δ*G*^‡^_298_) for this reaction pathway is only 21.7 kcal mol^−1^, and radical 4 lies 7.2 kcal mol^−1^ below the MF + ˙OH pair. Thus, making the reaction spontaneous at room temperature. From radical 4 into the final products, the Δ*G*^‡^_298_values are also not exceedingly high; 29.8 and 28.1 kcal mol^−1^, for TS5 and TS6, respectively, and the overall reaction is spontaneous with Δ*G*_298_ of −22.4 and −23.9 kcal mol^−1^. These values once again suggest favorable reactions between MF and ˙OH radicals, which agrees with experimental results.

**Scheme 4:**
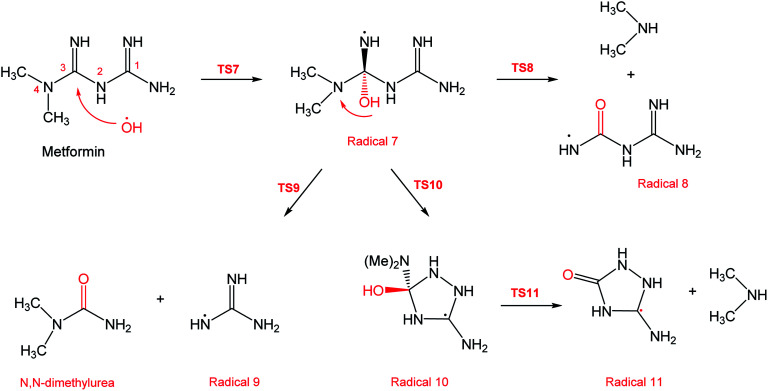
Reaction mechanism C initiated by ˙OH radical attack on C3 in metformin.

In a similar manner to the attack on C1, an ˙OH primary attack on C3 of MF leads to the formation of radical 7, as illustrated in Scheme 4. In this reaction pathway, the free radical 7 can decompose into dimethylamine (DMA) and radical 8 through TS8, or to *N*,*N*-dimethylurea (DMU) and radical 9, which can abstract hydrogen and form guanidine, through TS9. Both DMA and DMU were repeatedly detected during our GCMS analysis. Another possible pathway is the cyclization of radical 7 into 10 through TS10, which proceeds to radical 11 and DMA through TS11. Optimized geometries for the species involved in this mechanism are shown in Fig. S4 in the ESI.† The structure of radical 11 is similar to one of the products detected by our GCMS, 1-methyl-4-methylaminocytosine. Judgment on GCMS peaks is based on the fragmentation patterns, mass losses associated with the parent molecule, as well as similarly searches with library data. Because the two structures have similar mass spectra, it might be possible that the neutral form of radical 11 is formed during the oxy-cracking experiments. Furthermore, structure 8 is the radical form of guanylurea, which was detected in small amounts during the GCMS analysis (not shown in Fig. 3). It was also reported that guanylurea is the main product formed by the degradation of MF in wastewaters.^16^ These observations support the reaction mechanism proposed in Scheme 4.

The energy diagram of this mechanism is shown in Fig. 7. The first activation barrier, in terms of Δ*G*^‡^_298_, is estimated at 22.1 kcal mol^−1^. Therefore, attacks of ˙OH on C1 and C3 are mutually equal. Radical 7 is also more stable than the MF + ˙OH pair, similar to radical 4. The cyclization step to form radical 10 from radical 7, through TS10 (30.6 kcal mol^−1^), is less favorable than the decomposition steps through TS8 (18.8 kcal mol^−1^) and TS9 (1.5 kcal mol^−1^). Also the reaction is endergonic at (Δ*G*_298_ = 16.8 kcal mol^−1^), while the formation of DMA and 8, or DMU and 9 are exergonic (−18.7 and −25.4 kcal mol^−1^, respectively).

**Fig. 6:**
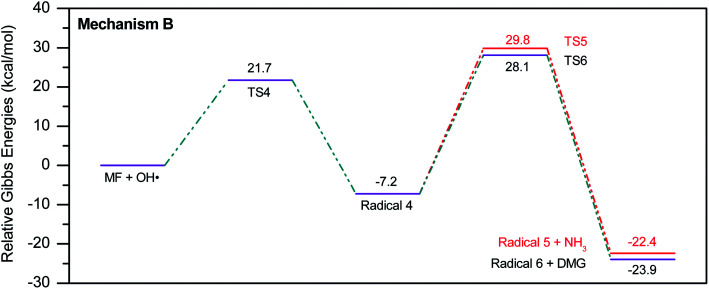
Potential energy level diagram for reaction mechanism B, initiated by ˙OH radical attack on C1 in metformin. Energies represent relative Gibbs free energies in kcal mol^−1^ at 298 K and 1 atm (ZPE corrections are included).

**Fig. 7:**
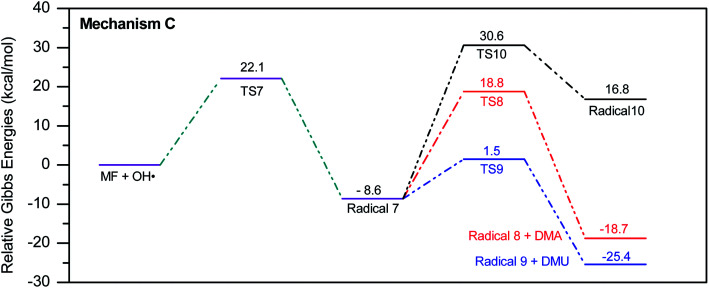
Potential energy level diagram for reaction mechanism C, initiated by ˙OH radical attack on C3 in metformin. Energies represent relative Gibbs free energies in kcal mol^−1^ at 298 K and 1 atm (ZPE corrections are included).

By looking at Fig. 6 and 7, it is clear that the formation of both DMG and DMU is the most favorable kinetically and thermodynamically. With relatively low activation barriers (Δ*G*^‡^_298_) and negative Δ*G*_298_ values. No wonder why these two products were among the most dominant during the oxy-cracking reaction of MF.

**Scheme 5:**
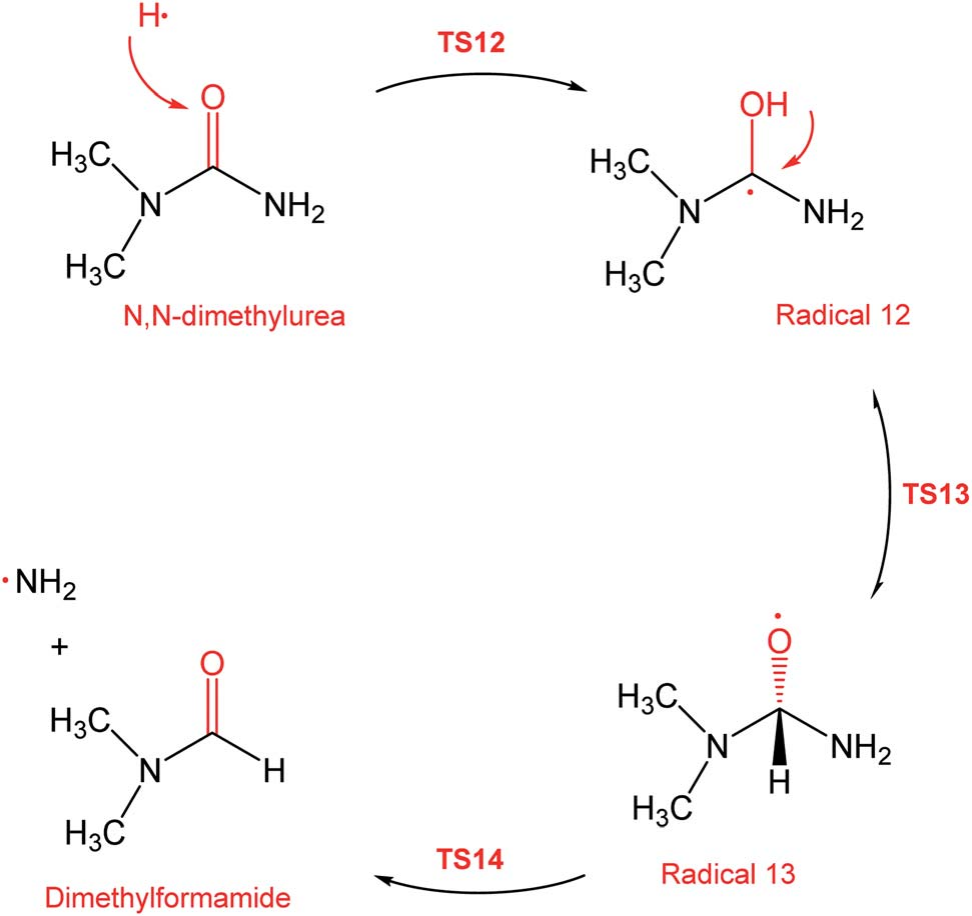
Reaction mechanism D, the formation of dimethylformamide out of dimethylurea.

**Fig. 8:**
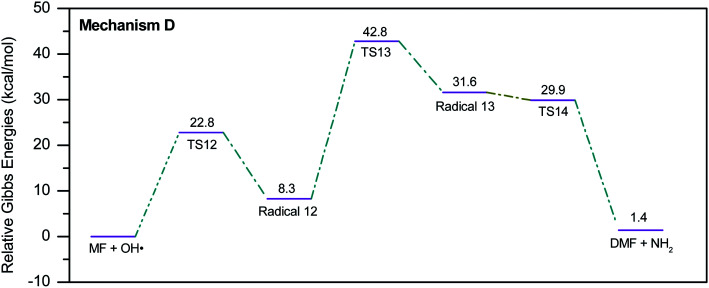
Potential energy level diagram for reaction mechanism D, the formation of dimethylformamide out of dimethylurea. Energies represent relative Gibbs free energies in kcal mol^−1^ at 298 K and 1 atm (ZPE corrections are included).

During the GCMS analysis of the oxy-cracked MF sample, dimethylformamide (DMF) was observed as a major product. To account for its formation, the mechanism illustrated in Scheme 5 is proposed. Starting from dimethylurea (DMU), an abstraction of H radical, which is generated under the oxy-cracking conditions, would lead to the formation of radical 12 through TS12. This is followed by isomerization to radical 13 through TS13, ending with a loss of ˙NH_2_ radical and DMF. Optimized geometries for the transition states and the free radicals of this mechanism are included in the ESI (Fig. S5†). As shown in the potential energy diagram in Fig. 8, the first energy barrier from the DMU and H˙ pair to TS12 is 22.8 kcal mol^−1^. The whole process is slightly endergonic by 1.4 kcal mol^−1^, which makes it favorable at higher temperatures. Particular, at the operating temperature of oxy-cracking that exceeds 150 °C.

## 4 Conclusions

Oxy-cracking of the antidiabetic drug metformin was investigated to assess its conversion in wastewater treatment plants. The experiments were performed under an excess pressure of oxygen and in the temperature range of 150–220 °C. A basic medium (pH > 8) was maintained in order to solubilize the acidic products and prevent corrosion to the reactor. Under these conditions, the hydroxyl (˙OH) radical can play a major role in initiating the free radical reactions causing the oxidation and decomposition of metformin. Online gas chromatography monitoring of the gases evolved during the reaction confirmed minimum evolution of CO_2_. No harmful gases such as CO or NO_*x*_ were observed. Total organic carbon analysis showed a slight decrease in the oxygenated hydrocarbons formed out of the oxy-cracking in the liquid phase, and thus avoiding complete oxidation onto CO_2_ and other gases. Nevertheless, an increase in the amount of evolved CO_2_ to the gas phase was observed at a higher temperature (220 °C) and longer reaction time (2 h). An GCMS analysis was performed for the oxy-cracked samples of MF after liquid–liquid extraction using dichloromethane. In addition to ammonia, mono- and dimethylamines, the small verity of organic compounds were detected. Among them are, *N*-nitrosodimethylamine (NDMA), *N*,*N*-dimethyl formamide (DMF), *N*,*N*-dimethylurea (DMU), dimethylguanidine (DMG), and hydroxyacetonitrile. The quantity and the signal intensity of the formed products were decreasing as the oxy-cracking conditions become harder. This infer that higher temperature and longer reaction times drive these small organic materials to completely oxidize into CO_2_, in agreement with the TOC analysis. Hence, one need to pay attention to optimize the oxy-cracking conditions in order to minimize CO_2_ conditions. The best operating conditions for the oxy-cracking of MF was found to be at 180 °C and reaction time of 2 h. Finally, a full reaction mechanism was proposed for the oxy-cracking reaction initiated by ˙OH radical attacks on MF. All transitions of the reaction mechanism were located successfully using high level *ab initio* calculations. The mechanism has accounted for the formation of many of the products that were observed during the GCMS analysis. Surprisingly, activation barriers in terms of free Gibbs energies of activation (Δ*G*^‡^_298_) were calculated to be as low as *ca.* 20 kcal mol^−1^, inferring very fast reactions between ˙OH radicals and the nitrogen-rich metformin molecule. Furthermore, most reaction pathways were found to be spontaneous at room temperature, and therefore, does so at higher temperatures.

## Conflicts of interest

There are no conflicts to declare.

## Supplementary Material

RA-009-C9RA01641D-s001
