# Supplementary material for: A combined experimental and density functional theory study of metformin oxy-cracking for pharmaceutical wastewater treatment
Source: RSC Adv. 2019 May 1;9(24):13403–13. doi: 10.1039/c9ra01641d (PMC9063927; doi:10.1039/c9ra01641d)
Supplement: RA-009-C9RA01641D-s001 [file RA-009-C9RA01641D-s001.pdf]

Electronic Supplementary Material (ESI) for RSC Advances.
This journal is © The Royal Society of Chemistry 2019

A Combined Experimental and Density Functional Theory Study of Metformin Oxy-Cracking for Pharmaceutical Wastewater Treatment

Ismail Badran[1,2], Abdallah D. Manasrah[1], Nashaat N. Nassar[1,]*

[1]Department of Chemical and Petroleum Engineering, University of Calgary, 2500 University Drive NW, Calgary, Alberta, Canada T2N 1N4

[2] Department of Chemistry, An-Najah National University, Nablus, Palestine

* Corresponding Author: Nashaat N. Nassar, Tel.: +1 403 210 9772; Fax: +1 403 210 3973, E-mail: nassar@ucalgary.ca

Supplementary Information

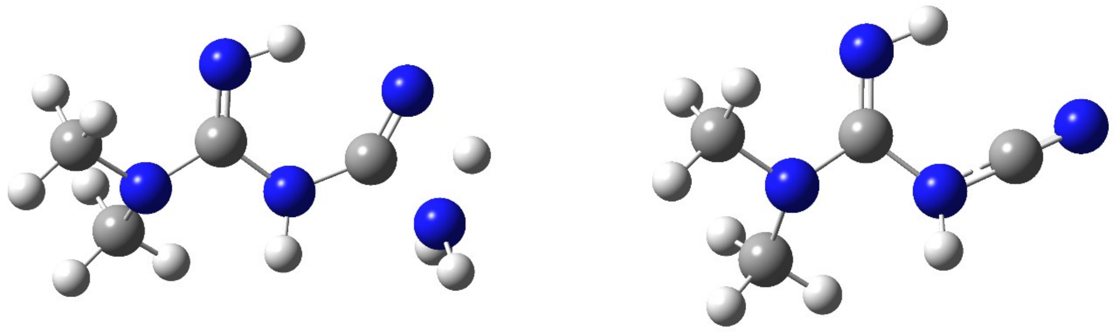

Figure S1: Optimized structures for a) the transition state for the 1,3-H shift in metformin leading to the formation of ammonia, and b) the product of this shift. Both obtained at B3LYP/6-31+G(d,p) level of theory. C atom, grey; N atom, blue.

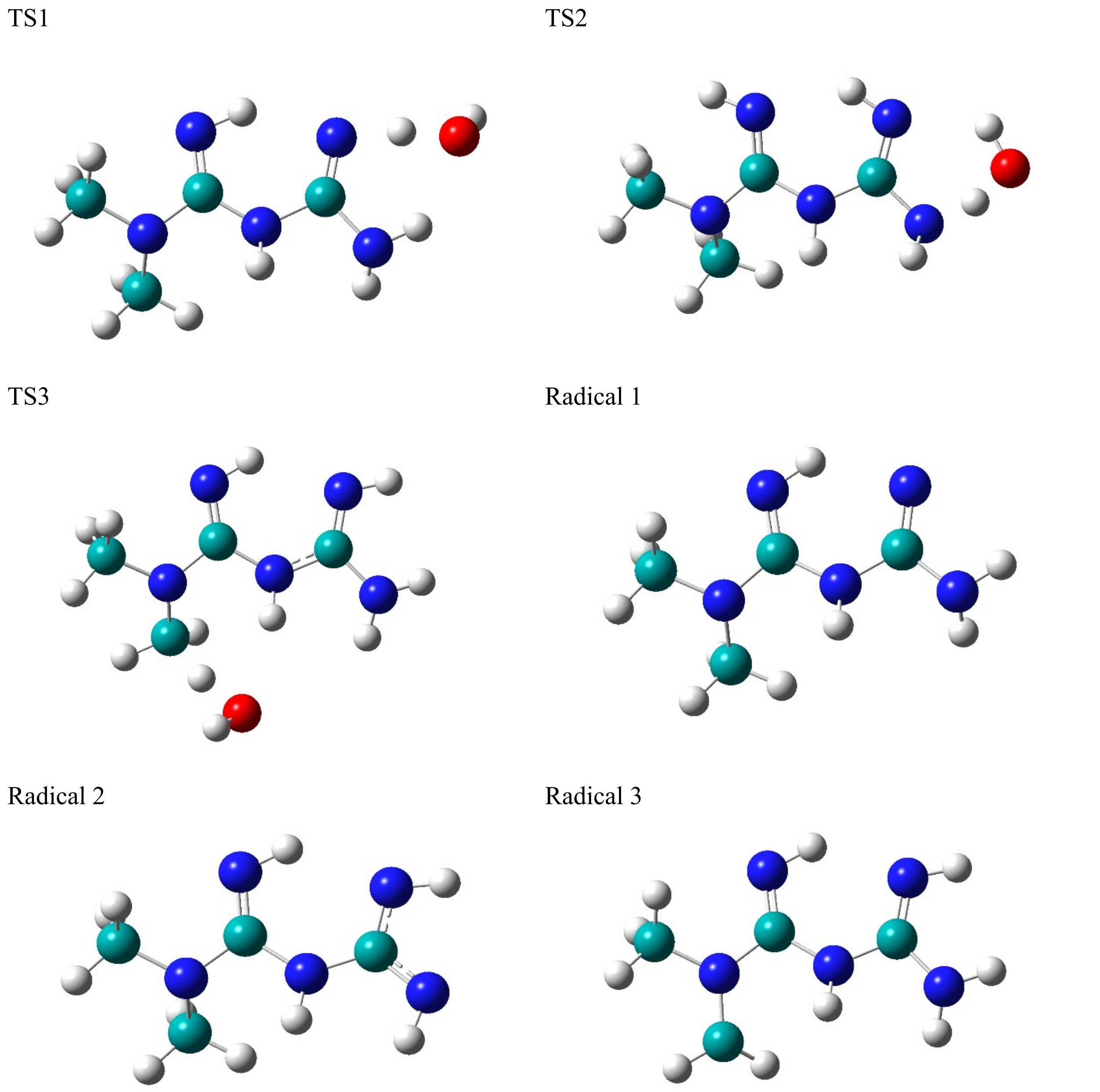

Figure S2: Optimized structures for the transition states and free radicals involved in reaction mechanism A, hydrogen abstraction by OH radical in metformin, obtained at B3LYP/6-31+G(d,p) level of theory. C atom, grey; N atom, blue; O atom, red.

TS4

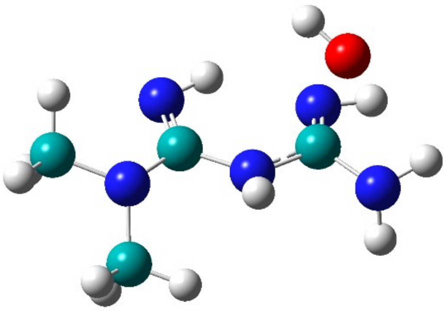

TS5

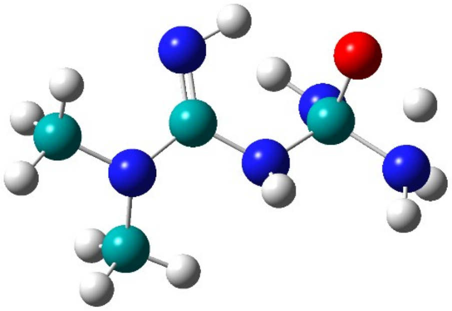

TS6

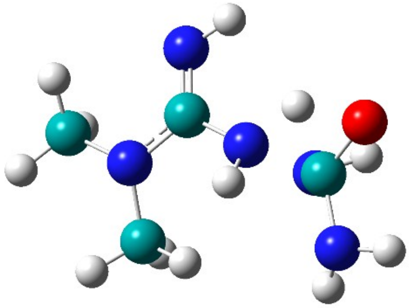

Radical 4

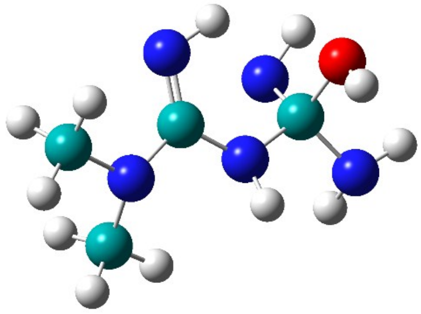

Radical 5

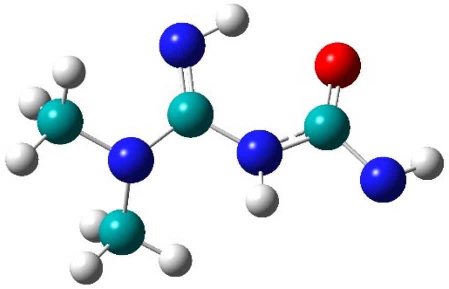

Radical 6

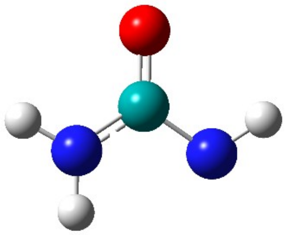

Dimethylguanidin

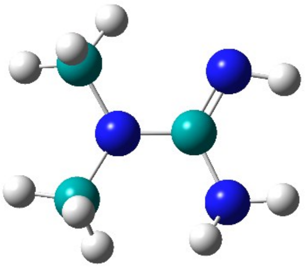

Figure S3: Optimized structures for the transition states and free radicals involved in reaction mechanism B initiated by OH radical attack on C1 in metformin, obtained at B3LYP/6-31+G(d,p) level of theory. C atom, grey; N atom, blue; O atom, red.

TS7

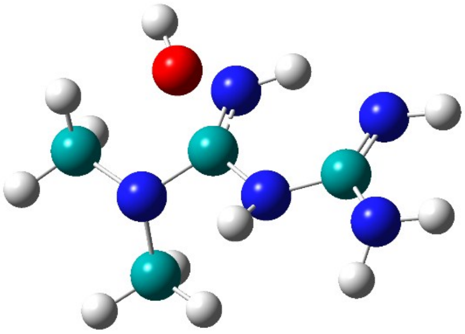

TS8

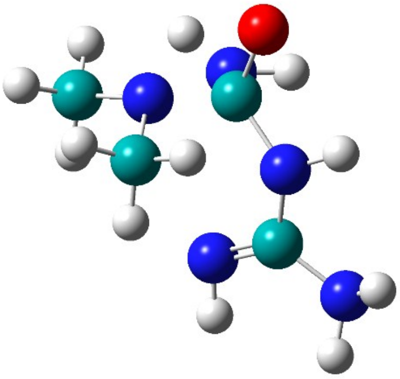

TS9

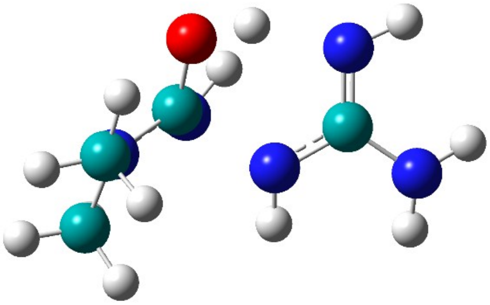

TS10

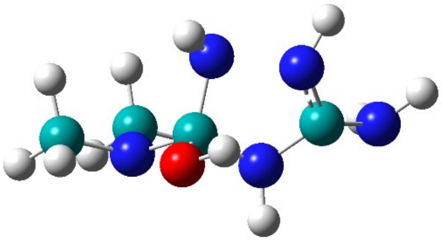

TS11

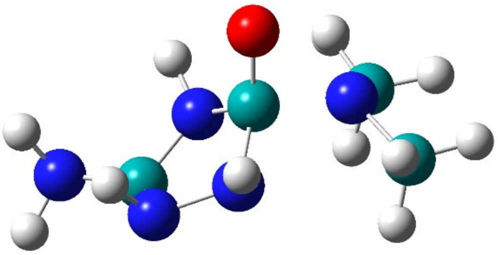

Radical 7

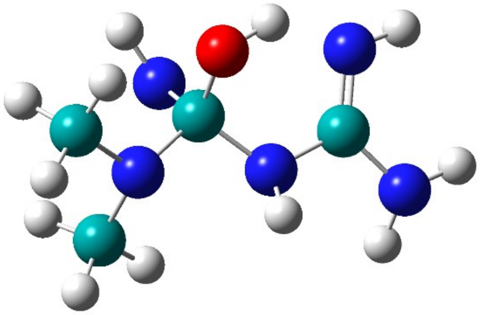

Radical 8

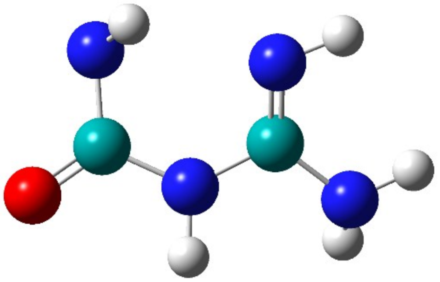

Radical 9

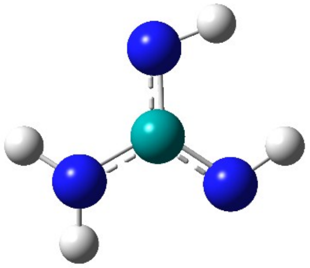

Radical 10

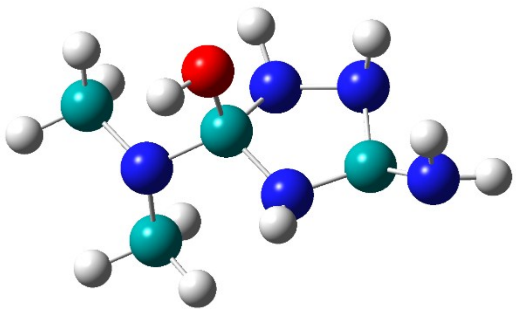

Radical 11

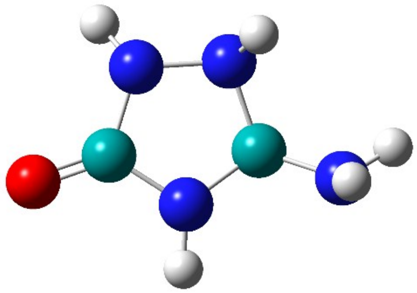

Dimethylamine

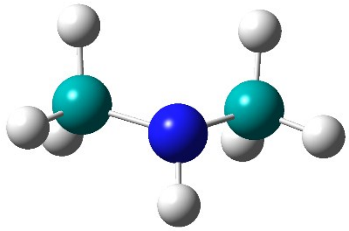

N,N,Dimethylurea

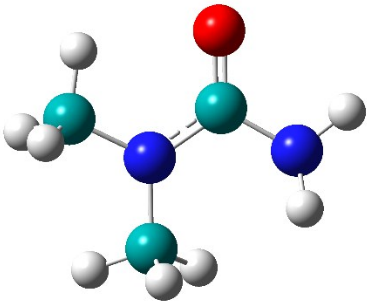

Figure S4: Optimized structures for the transition states and free radicals involved in reaction mechanism C initiated by OH radical attack on C3 in metformin, obtained at B3LYP/6-31+G(d,p) level of theory. C atom, grey; N atom, blue; O atom, red.

TS12

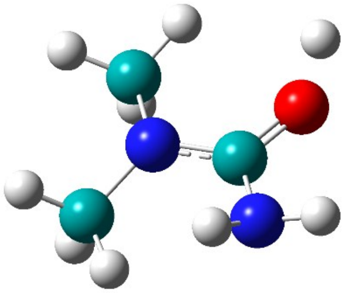

TS13

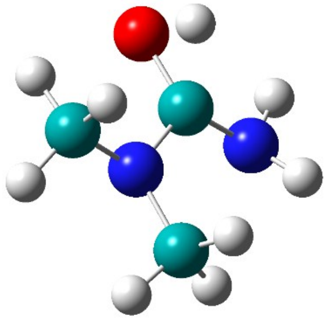

TS14

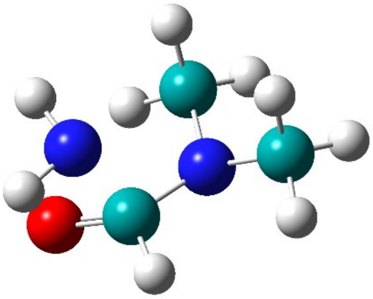

Radical 12

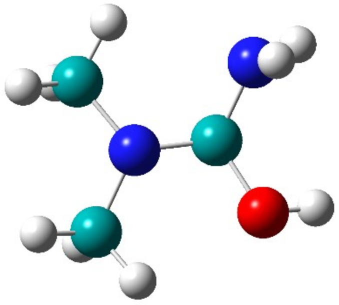

Dimethylformamide

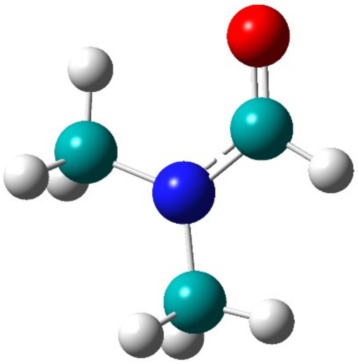

Figure S5: Optimized structures for the transition states and free radicals involved in reaction mechanism D, formation of dimethyl formamide out of dimethyl urea, obtained at B3LYP/6-31+G(d,p) level of theory. C atom, grey; N atom, blue; O atom, red.
